# Ocular blood flow values measured by laser speckle flowgraphy correlate with the postmenstrual age of normal neonates

**DOI:** 10.1007/s00417-016-3362-6

**Published:** 2016-04-27

**Authors:** Tadashi Matsumoto, Takashi Itokawa, Tomoaki Shiba, Yuuji Katayama, Tetsushi Arimura, Kotaro Hine, Norio Mizukaki, Hitoshi Yoda, Yuichi Hori

**Affiliations:** 1 Department of Ophthalmology, School of Medicine, Toho University, 6-11-1 Omori-Nishi, Ota-ku, Tokyo, 143-8541 Japan; 2 Department of Neonatology, School of Medicine, Toho University, Tokyo, Japan

**Keywords:** Laser speckle flowgraphy, Optic nerve head, Ocular blood flow, Normal neonate, Postmenstrual age, LSFG-baby

## Abstract

**Purpose:**

To evaluate the relationships between optic nerve head (ONH) blood flow by laser speckle flowgraphy (LSFG), and postmenstrual age and body weight in normal neonates.

**Methods:**

During their normal sleep, we studied 24 infants (postmenstrual age, 248–295 days) whose ocular blood flow could be measured three consecutive times. While the subjects slept in the supine position, three mean blur rate (MBR) values of the ONH were obtained: the MBR-A (mean of all values), MBR-V (vessel mean) and MBR-T (tissue mean) in the ONH. With regard to eye diseases, no retinopathy of prematurity (ROP) was observed, and no severe systemic diseases requiring treatment were noted in the subjects. Pearson’s correlation coefficients were used to determine the relationship between the MBR-A, −V, −T and postmenstrual age (days) and body weight (g).

**Results:**

Postmenstrual age was significantly correlated with MBR-A (*r* = 0.64, *p* = 0.0007), MBR-V (*r* = 0.62, *p* = 0.0012) and MBR-T (*r* = 0.62, *p* = 0.0012). However, the body weight was not correlated with the MBR (MBR-A: *r* = 0.37, *p* = 0.07, MBR-V: *r* = 0.31, *p* = 0.14, MBR-T: *r* = 0.38, *p* = 0.06).

**Conclusions:**

Our results clarified that the values of ocular blood flow measured by LSFG correlate with the postmenstrual age of normal neonates.

## Introduction

Laser speckle flowgraphy (LSFG) can be used to noninvasively measure ocular blood flow [1–4]. LSFG is based on the changes in the speckle pattern of laser light reflected from the eye [5], and since LSFG is dependent on the movement of erythrocytes in the retina and choroid [6], it can measure the relative velocity of the erythrocytes’ mean blur rate (MBR). LSFG is used for research on various diseases in adult patients, such as glaucoma [7, 8], retinal vein occlusion [9] and diabetic retinopathy [10]. It is also used in research related to aging and retinal blood flow changes [11–13]. Ocular diseases in neonates such as the retinopathy of prematurity (ROP) changes in retinal circulation occur with dilation and tortuosity of retinal blood vessels [14].

Until now, studies of the neonatal ophthalmic artery (OA) and central retinal artery (CRA) have been made using Color Doppler imaging (CDI) [15–20]. However, there has been no study taking direct measurements of the intraocular blood flow, and the relation between ROP and the ocular blood flow is still unknown. We have nevertheless speculated that the relationships between the ocular blood flow and neonatal ocular diseases could be clarified by researching retinal hemodynamics in neonates. We therefore measured the ocular blood flow in neonates using a version of LSFG modified for use in neonate patients (i.e., the LSFG-baby system), and we observed that the reproducibility of the results obtained with LSFG-baby is good [21].

The details of neonatal ocular blood flow changes are not known, but it is known that (1) the amount of general circulation in neonates is related to the neonatal body weight (g), and (2) the cerebral blood volume (CBV) is related to the neonate’s postmenstrual age (days) [22]. For this reason, we also considered the possibility that the neonatal ocular blood flow is correlated with changes in body weight and postmenstrual age, and we conducted the present study to test this possibility.

We measured the ocular circulation of the optic nerve head (ONH) in normal neonates using LSFG-baby, and we assessed the relationships between these blood flow values and the body weight and postmenstrual age. Our findings thus define the standard values of LSFG and indices in the early neonatal period, and our results provide the first step in ocular circulation studies of ROP.

## Subjects and methods

### Subjects

The subjects for our investigation were non-incubator neonates who were not on respirators. For each subject, an ophthalmologist had requested an examination due to suspicion of ROP or other diseases between March 2015 and December 2015 at the Toho University Omori Medical Center. We studied 24 neonates (24 eyes) during sleep in whom the circulation could be measured three times consecutively at the initial test (11 males, 13 females; postmenstrual age: 248–295 days, i.e., 34–42 weeks). With regard to eye diseases, no ROP was identified, and no severe systemic diseases requiring treatment were noted in the subjects.

This study was conducted in accord with the principles laid out in the Declaration of Helsinki, and the data analysis was approved by the ethical review committee of Toho University (nos. #26-96, #27-19).

### LSFG measurement

**Fig. 1:**
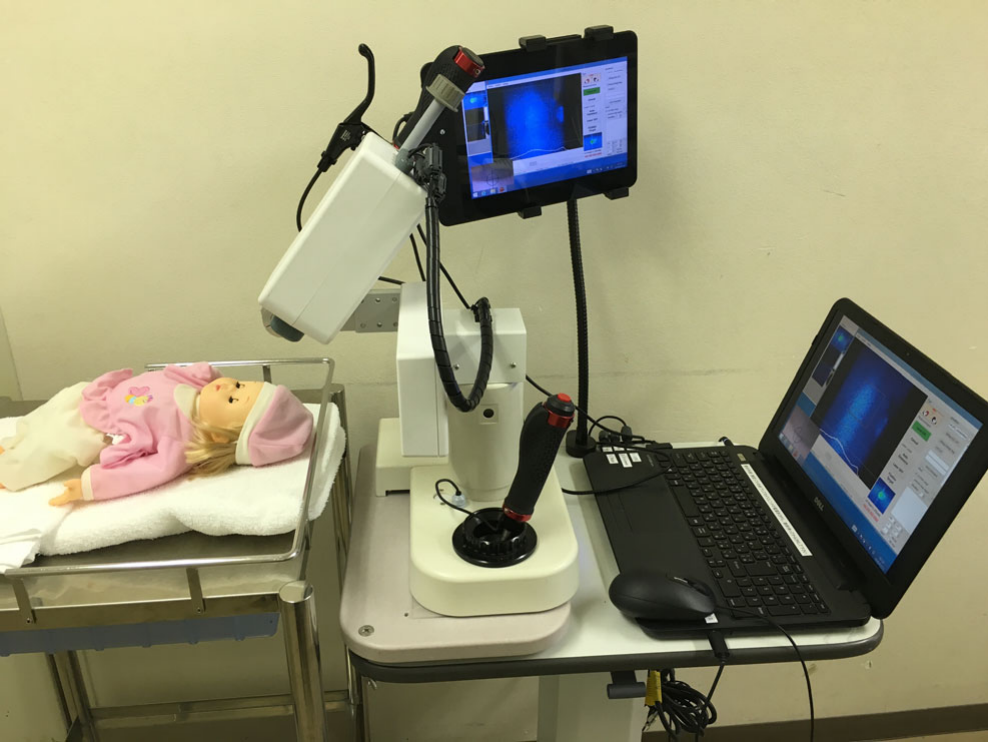
The LSFG-baby system. Examination of a neonate in the supine position. The system’s camera is set on a tilting stage with two axes (φ and θ) and x–y stages to adjust the field of view

Measurements were obtained using the ‘LSFG-baby’ system [21], which is a version of the commercially available LSFG-NAVI system (Softcare, Fukuoka, Japan) modified in such a manner that the testing can be performed with the neonate in the supine position (Fig. 1). The testing followed previously reported methods [21]. Briefly, pupillary dilation was achieved with 2.625 % phenylephrine hydrochloride, 0.125 % tropicamide and 0.25 % cyclopentolate, after which the ONH was imaged for 3 sec. Testing was performed while the infant was sleeping to measure his or her circulation during rest. Without the use of a lid speculum, the tester gently held the infant’s eyelid open with a finger during the examination. All measurements were performed by the same tester. The testing was concluded within 10 min, and measured only the left eye. After testing, the images were confirmed, and results that were significantly out of focus, or for which only two heart beats or less were measured, were excluded.

### Measurements of systemic parameters and other ocular parameters

The following parameters were measured: systolic blood pressure (SBP, mmHg), diastolic blood pressure (DBP, mmHg), pulse pressure (mmHg), heart rate (beats per min, bpm), and intraocular pressure (IOP, mmHg) measured by a Tono-Pen Avia (RE Medical, Osaka, Japan). We calculated the mean arterial blood pressure (MABP, mmHg) and ocular perfusion pressure (OPP, mmHg) with the following formulas. The MABP was determined by the formula: DBP + (SBP − DBP) / 3). The OPP was defined as: (2/3MABP) − IOP. All parameters were evaluated after the LSFG measurements were obtained.

### Analysis of the MBR values of the ONH

**Fig. 2:**
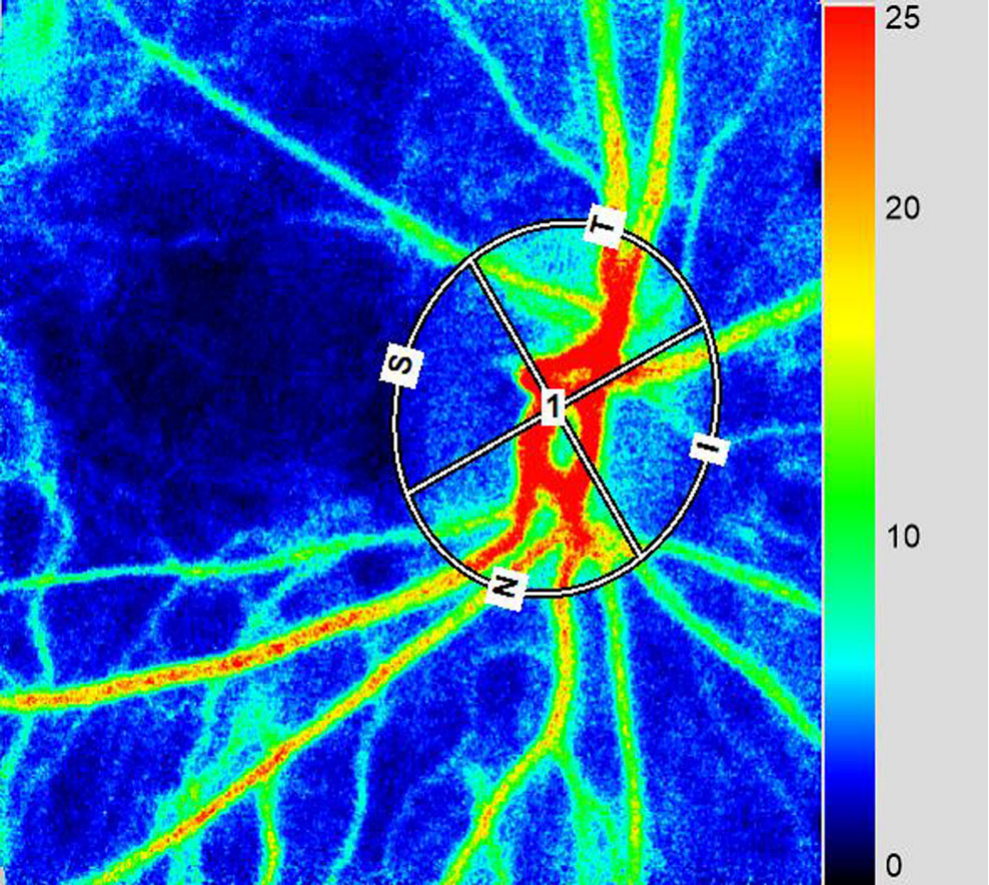
In the color-coded maps, *red* indicates high blood flow and *blue* indicates low blood flow. The image is observed indirectly

We analyzed the MBR by setting a rubber band on the ONH [23] (Fig. 2). The three MBR parameters of MBR-A (the mean of all values), MBR-V (the vessel mean) and MBR-T (the tissue mean) were analyzed by the LSFG Analyzer software (Softcare). Each result was a mean of three measurements. For the evaluation of the reproducibility of the measurement method, the analysis was performed using the MBR of the ONH with which the coefficient of variation (CV) and intraclass correlation coefficient (ICC) were measured three consecutive times.

### Statistical analysis

Data are presented as the mean±standard deviation (SD) for the continuous variables. Pearson’s correlation coefficients were used to determine the relationship between the MBR-A, MBR-V, MBR-T and postmenstrual age, body weight, and other systemic and ocular parameters. *P* values < 0.05 were considered significant. The data were analyzed using the 12.1.0 version of the statistical software JMP (SAS, Cary, NC).

## Results

Table 1 shows the characteristics of the 24 subjects. Table 2 shows the CVs and ICCs for the MBR of the ONH values of the 24 subjects. All CVs from these patients were < 10 %, and all ICC results were > 0.8. The MBR-A was 11.2 ± 3.3 (range 5.7–17.7), the MBR-V was 21.5 ± 5.9 (range 11.7–30.5) and the MBR-T was 7.9 ± 2.5 (range 3.5–14.2).

**Table 1:** Characteristics of the 24 neonates

	Mean±SD
Postmenstrual age (days)	267.5 ± 13.9
Gestational age (days)	237.7 ± 21.2
Chronologic age (days)	29.8 ± 17.2
Body weight (g)	2245.3 ± 387.3
Birth weight (g)	1867.1 ± 632.3
Heart rate (bpm)	140.0 ± 14.1
MABP (mmHg)	47.8 ± 5.3
IOP (mmHg)	15.4 ± 2.4
OPP (mmHg)	16.5 ± 4.0
Gender (m:f)	11:13

*MABP* mean arterial blood pressure, *IOP* intraocular pressure, *OPP* ocular perfusion pressure

**Table 2:** Coefficients of variation (CV) and intraclass correlation coefficient (ICC) for ONH blood flow values (*n* = 24)

	CV (%)	ICC	Mean±SD
MBR-A	8.3 ± 3.1	0.87	11.2 ± 3.3
MBR-V	9.8 ± 4.6	0.80	21.5 ± 5.9
MBR-T	9.2 ± 4.1	0.87	7.9 ± 2.5

*MBR* mean blur rate, *MBR-A* mean of all values, *MBR-V* vessel mean, *MBR-T* tissue mean

**Fig. 3:**
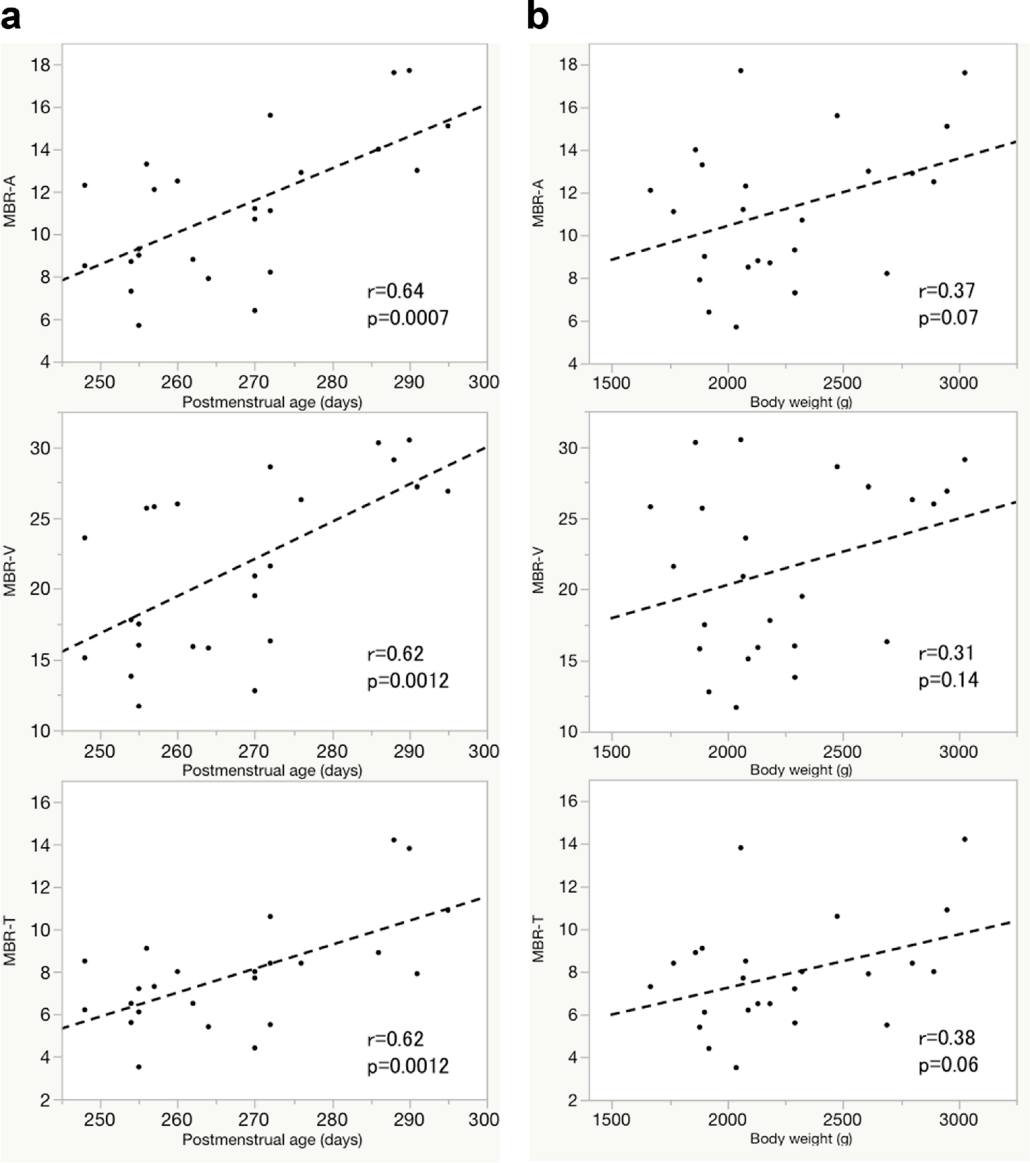
Correlations between MBR and postmenstrual age (days) or body weight (g). **a** Relationships between MBR (−A [all values’ mean], −V [vessel mean], −T [tissue mean]) and postmenstrual age. **b** Relationships between MBR (−A, −V, −T) and body weight

The Pearson’s correlation coefficients between the MBR of the ONH and postmenstrual age and body weight are given in Table 3. Postmenstrual age was significantly correlated with the MBR-A (*r* = 0.64, *p* = 0.0007), MBR-V (*r* = 0.62, *p* = 0.0012) and MBR-T (*r* = 0.62, *p* = 0.0012) (Fig. 3a). However, the body weight was not correlated with the MBR (MBR-A: *r* = 0.37, *p* = 0.07; MBR-V: *r* = 0.31, *p* = 0.14; MBR-T: *r* = 0.38, *p* = 0.06; Fig. 3b).

**Table 3:** Pearson’s correlation coefficients between MBR (−A, −V, and -T) and all parameters (*n* = 24)

Explanatory variable	MBR-A		MBR-V		MBR-T	
	*r*	*p*	*r*	*p*	*r*	*p*
Postmenstrual age (days)	0.64	0.0007	0.62	0.0012	0.62	0.0012
Gestational age (days)	0.23	0.29	0.22	0.31	0.21	0.32
Chronologic age (day)	0.24	0.25	0.24	0.26	0.24	0.26
Body weight (g)	0.37	0.07	0.31	0.14	0.38	0.06
Birth weight (g)	0.08	0.72	0.05	0.83	0.07	0.74
Heart rate (bpm)	0.02	0.94	−0.08	0.70	0.15	0.49
OPP (mmHg)	−0.13	0.55	−0.09	0.69	−0.15	0.47

*MBR* mean blur rate, *MBR-A* mean of all values, *MBR-V* vessel mean, *MBR-T* tissue mean, *OPP* ocular perfusion pressure

In addition, the following parameters were not correlated with any aspect of the MBR: gestational age, chronologic age, birth weight, heart rate and OPP.

## Discussion

This study is the first to examine the blood flow in normal neonates using LSFG. We measured the ocular blood flow in 24 neonates, and our findings revealed a correlation between the MBR of the ONH and postmenstrual age. No correlation was observed between the MBR and body weight, gestational age, chronological age, birth weight or OPP. The reproducibility of the results was excellent, with a CV of not more than 10 % and an ICC of not less than 0.8, as reported earlier [21].

The correlation between the volume of the general circulation of neonates and their body weight is generally understood, and the body weight is used for calculating the general circulation volume. However, it has been reported that the cerebral circulation volume expressed as CBV correlates more strongly with corrected age in days than with body weight [22]. The growth of the head, including the eyeballs, is generally completed sooner than the development of the whole body, which may be related to the results of this study. In other words, it is likely that the mean blur rate of the ONH is involved in eye development in terms of function due to changes in the age in days rather than changes in whole-body growth and circulation.

Earlier studies of ocular blood flow in neonates by CDI showed an increase in the blood flow velocity in the OA [15, 17] and CRA [17, 20] along with age in days. However, to our knowledge, there is no study on the correlation of these blood flow velocities with postmenstrual age or body weight. Further, CDI determines the absolute value of blood flow velocity, but this may not reflect the changes in the blood flow volume if the blood vessel diameter has changed.

On the other hand, in studies that used LSFG, since the blood flow value is determined by assigning a rubber band directly to the ONH [21], LSFG is thought to be advantageous for neonatal blood flow measurement. In addition, unlike CDI, LSFG does not provide the absolute value of the blood flow volume, and making comparisons between individuals with LSFG is said to be difficult [23]. However, Aizawa et al. [24] are of the opinion that the MBR of the ONH determined by LSFG and the values measured using the hydrogen clearance method correlate well under almost identical conditions, and therefore can be used for comparing the MBR of the ONH between individuals.

The MBR for the normal neonates’ ONHs in this study was lower compared to that for adults (age 60.4 ± 12.5 years, *n* = 93) [25] who we examined: MBR-A, 21.9 ± 3.8; MBR-V, 42.1 ± 4.8; MBR-T, 11.9 ± 1.9. However, due to major differences between adults and neonates with regard to heart rate, blood pressure, OPP, and eye structure, a simple comparison is difficult.

This study has some limitations. First, examinations of the neonates, unlike adults, are difficult. The reproducibility of the examinations is also slightly inferior compared to the results of adults [7]. Second, the pupil diameter of a sleeping neonate is small, and therefore the examination is impossible without mydriasis. Third, due to the noninvasive nature of this study with natural sleeping neonates, observations of neonates who are under artificial ventilation in an incubator or infants who grew and were discharged from the hospital would be difficult with the LSFG-baby system. However, with further improvements to LSFG measurement devices, the number of test subjects will increase along with the accumulation of data, and studies in such subjects could become possible.

Here, we measured the mean blur rate and the neonatal ocular circulation. The results of our study suggest that neonatal ocular circulation measurements could become an indicator of eyeball function and retinal circulation growth, and our findings a first step in the study of the correlation of eye diseases and systemic diseases with ocular blood flow in neonates.
